# A score without diffusion capacity of the lung for carbon monoxide for estimating survival in idiopathic pulmonary fibrosis

**DOI:** 10.1097/MD.0000000000020739

**Published:** 2020-06-19

**Authors:** Cesar Yoshito Fukuda, Maria Raquel Soares, Carlos Alberto de Castro Pereira

**Affiliations:** Universidade Federal de São Paulo, Escola Paulista de Medicina, São Paulo, Brazil.

**Keywords:** diffusion capacity of the lung for carbon monoxide, exercise desaturation, idiopathic pulmonary fibrosis, interstitial lung diseases, survival

## Abstract

Prediction models for survival at baseline evaluation have been proposed in idiopathic pulmonary fibrosis (IPF) but include diffusion capacity of the lung for carbon monoxide, a test not available in many places. The aim of the present study was to develop a simple new mortality risk scoring system for patients with IPF at initial evaluation without diffusion capacity of the lung for carbon monoxide measurement.

A total of 173 patients, 72% males, mean age 70 years, 64% smokers/ex-smokers, were included in a retrospective study. The diagnosis was made by surgical lung biopsy in 40 (23%); in the remaining patients, a usual interstitial pneumonia pattern was present in high-resolution computed tomography. Patients with forced expiratory volume in 1 second/forced vital capacity ratio (FEV_1_/FVC) <0.70 were excluded. Dyspnea was evaluated by magnitude of task on the Mahler scale (Chest 1984). Peripheral oxygen saturation was measured by oximetry at rest and at the end of a 4 minutes step test or a 6-minute walk test.

At the end of the follow-up period, 154 (89%) of the patients had died. Based on the univariate Cox proportional-hazards model, survival (*P* ≤ .10) was related directly to the dyspnea score, presence of cough, lower values of FVC% and FEV_1_%, lower rest and oxygen desaturation during exercise, and greater FEV_1_/FVC. By Cox multivariate analysis, the results remained correlated to the survival dyspnea score, FVC%, and exercise peripheral oxygen saturation. A score, using these variables, was developed and was able to discriminate among 3 groups, with high, low, and intermediate survival curves.

A prognostic score, taking into account dyspnea, FVC%, and oxygen desaturation during exercise, can estimate survival in IPF.

## 1 Introduction

Idiopathic pulmonary fibrosis (IPF) is defined as a specific form of chronic, progressive, fibrosing, interstitial pneumonia of unknown cause, occurring primarily in older adults; it is limited to the lungs and associated with the histopathologic and/or radiologic pattern of usual interstitial pneumonia (UIP).^[1]^

The median survival of IPF, before modifier treatments, was between 2 and 4 years after the diagnosis.^[2]^ Most of the patients demonstrated a gradual progression over many years, but some patients can experience a rapid decline in lung function or remain stable for long periods.^[2]^ This variability leads to difficulties in developing estimates for prognosis; nevertheless, several prognostic scores have been suggested for IPF. These are relevant for clinical decision making, such as for the timing of lung transplantation, and simplifying clinical trial designs.^[3]^

Many individual clinical variables have been shown to predict survival in IPF, such as age, gender, dyspnea, baseline forced vital capacity (FVC), diffusion capacity of the lung for carbon monoxide (D_L_CO), forced expiratory volume in 1 second/forced vital capacity (FEV_1_/FVC) ratio, oxygen desaturation during exercise (ExSpO_2_) and others.^[4–10]^ Composite scores can estimate survival better in IPF, but different variables are included in these models. D_L_CO appears to be the most reliable predictor of survival at baseline and is included in the majority of them. However, D_L_CO measurement is not available in many places, especially in developing countries.

The aim of the present study was to develop a simple prognostic score for IPF in places where D_L_CO measurements are not available, using a well-defined, retrospective cohort of patients with IPF and long follow-up.

## 2 Methods

“Ethical approval for this study (Ethical Committee CAAE N°: 07397918.4.0000.5505) was provided by Comitê de Ética em Pesquisa - Unifesp, São Paulo, on 04 April 2019.”

### 2.1 Study population

The study included retrospectively identified patients with IPF from 3 reference centers for interstitial lung diseases (ILD) in São Paulo.

The patients were identified through a review of medical records obtained between June 4, 1993, and December 30, 2016.

The diagnosis of IPF was based on the following: the presence of a definitive high-resolution computed tomography (HRCT) pattern and age >50 years; a definitive UIP pattern from a surgical lung biopsy (SLB) in those with a possible IPF pattern on HRCT; or a UIP pattern both on HRCT and in SLB.^[1]^ All cases were reviewed by experienced pulmonologists and radiologists, and all biopsies were reviewed by lung pathologists with extensive experience in ILD.

Patients with an airflow obstruction (FEV_1_/FVC ratio <0.70), any evidence of disease that could result in UIP,^[1]^ and on treatment with pirfenidone or nintedanib, were excluded.

In cases with a resting SpO_2_ ≤88%, exercise was not performed, and ExSpO_2_ was presumed to be <85%. Survival data was analyzed by Kaplan–Meyer curves and Cox analysis.

The informed consent was waived because this is a retrospective study and involved no more than minimal risk to the participants.

### 2.2 Predictor variables

Duration of symptoms; gender; age at symptom onset; smoking status; symptoms of gastroesophageal reflux disease (heartburn, regurgitation), dyspnea, cough, crackles, finger clubbing, and presence of honeycombing or emphysema on HRCT; pulmonary functional variables (FVC, FEV_1_, FEV_1_/FVC ratio, and D_L_CO); and peripheral oxygen saturation (SpO_2_) at rest and after exercise were recorded using a systematic protocol. The patients were categorized as nonsmokers or smokers (current or former smokers).

Dyspnea was assessed by magnitude of task of the basal dyspnea index.^[11]^ Total basal dyspnea index score was not considered because functional impairment and magnitude of effort do not involve the same activities in different patients. SpO_2_ was measured by oximetry at rest and at the end of a 4-minute step test or a 6-minute walk test (6 MWT).^[12,13]^ SpO_2_ in these 2 tests has similar prognostic value in IPF.^[14,15]^ No supplemental oxygen was used during exercise, and SpO_2_, measured by digital oximetry (Nonin) at rest and at the end of exercise (ExSpO_2_), were evaluated at initial visits.

Pulmonary function tests were conducted according to standard criteria.^[16]^ The predicted values for spirometry were those derived from the Brazilian population.^[17]^ The decision of whether to provide specific treatment was made by individual clinicians.

### 2.3 Statistical analysis

To estimate the sample size for the Cox models, a minimum of 10 outcome events should be present per predictor variable.^[18]^ Possible categorical predictors were age, gender, dyspnea,^[4–6]^ FVC,^[4–10]^ FEV_1_/FVC,^[7]^ and ExSpO_2_ ≤88%.^[14,15]^

Based on the analysis of a previous study conducted in our center,^[19]^ approximately 120 cases should be included to obtain 60 deaths in a study with similar duration.

Analyses were completed using IBM SPSS, version 22. The values were expressed as count, percentage, mean, median, and standard deviation.

Group comparisons were made using unpaired tests (for normally distributed, continuous variables).

Correlations were calculated using Pearson coefficient.

Survival time was calculated from the date of diagnosis to death or lung transplantation (n = 2) or loss of follow-up. Survival status was obtained from telephone interviews and/or medical records in December 2016. In this study, all-cause mortality was evaluated.

The effect of each potential explanatory variable, expressed in continuous or categorical values, on the hazard function was calculated by univariate analysis, using a Cox proportional-hazards regression. To avoid multicollinearity, only one of the highly correlated variables (Pearson correlation coefficient ≥0.6) was entered in the multivariate model. Candidate variables with *P*-values of <.10 in a univariate analysis were then transformed into categorical variables.

Thresholds for physiological variables were based on previously published values,^[1,2,8,15,19,20]^ receiver operating characteristic points with greater sums of sensitivity and specificity, and the greatest log-rank in Kaplan–Meier analysis. The categorical variables to be included in the final model were selected by Cox multivariate analysis. Outliers were identified by SPSS and excluded.^[21]^ The results were summarized as hazard ratios (HRs), which represented the relative risk of death as a result of a specific characteristic during the observation period. Each predictor variable was categorized as 0, 1, or 2, and survival curves were compared among the summed final scores, using Kaplan–Meier curves.

The overall performance of the risk scoring system was quantified by the C-statistic.^[22]^

## 3 Results

A total of 180 patients were evaluated. Seven outliers were identified and excluded from the subsequent analyses, 2 with FVC >120% predicted, 2 with SpO_2_ at exercise <70%, 1 with D_L_CO <15%, one with D_L_CO = 100%, and 1 with an unexpected survival time of 163 months.

A total of 173 patients with IPF were included in the final analysis. Their baseline characteristics and clinical and physiological data are summarized in Table 1.

**Table 1:**
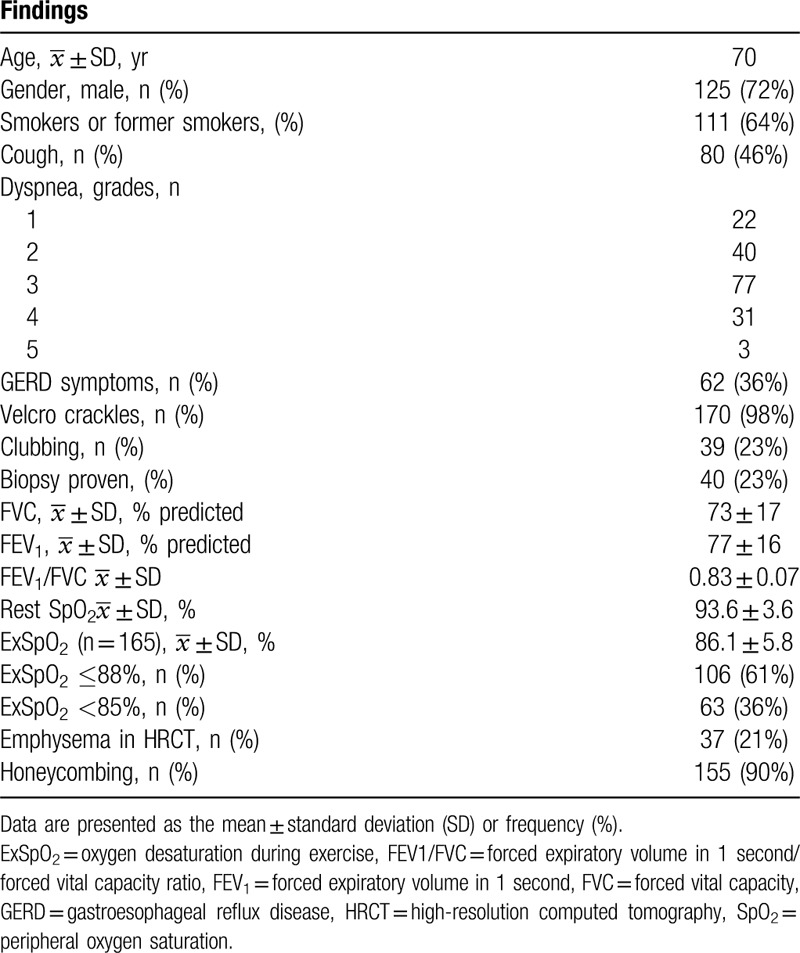
General findings in 173 patients with idiopathic pulmonary fibrosis.

The diagnosis was made by SLB in 40 (23%) cases, and in the remaining cases, a UIP pattern was present in HRCT. All patients who had no honeycombing in HRCT underwent SLB.

Most patients were male, with a mean age of 70 years (range 49–87 years), and 64% were smokers or former smokers. Based on FVC%, the restriction was typically mild (FVC = 73% ± 17%).

The median follow-up time was 42 months. The median survival was 43 months (95% confidence interval [CI]: 36–50 months). At the end of the follow-up period, 154 (89%) of the patients had died. All patients, except 3, died from IPF or related complications (2 died from lung cancer). Two were censored due to lung transplantation.

Based on the univariate Cox proportional hazards model, survival (*P* ≤ .10) was related directly to the dyspnea score, presence of cough, lower values of FVC% and FEV_1_%, lower rest and ExSpO_2_, and greater FEV_1_/FVC (Table 2). By Cox multivariate analysis, (forward Wald) survival remained correlated to dyspnea score, FVC percentage, and exercise SpO_2_ (Table 3). When age and gender were forced in the model, the results did not change.

**Table 2:**
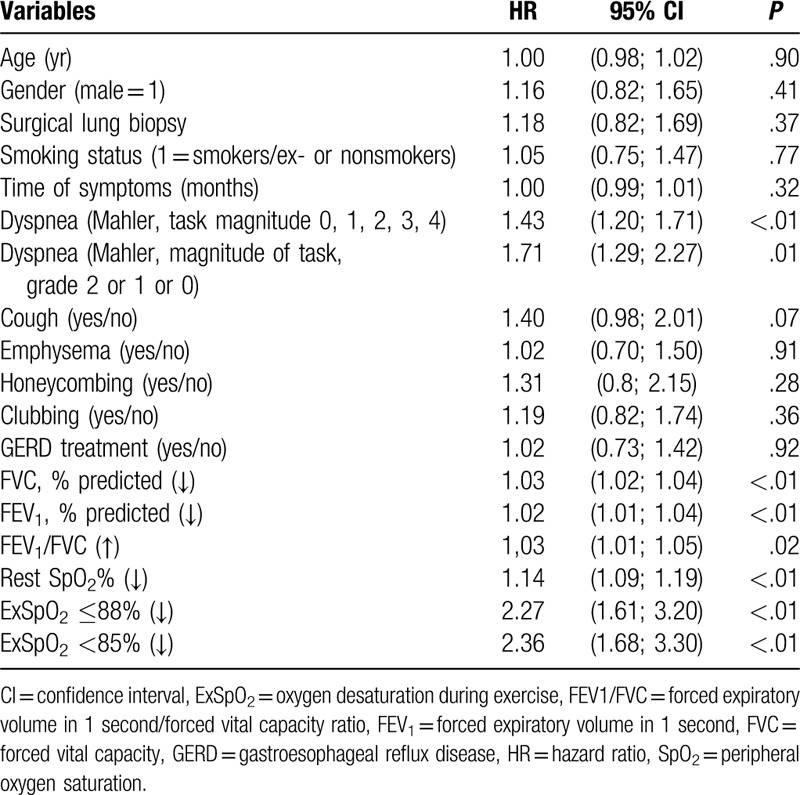
Univariate analysis: clinical and functional variables.

**Table 3:**
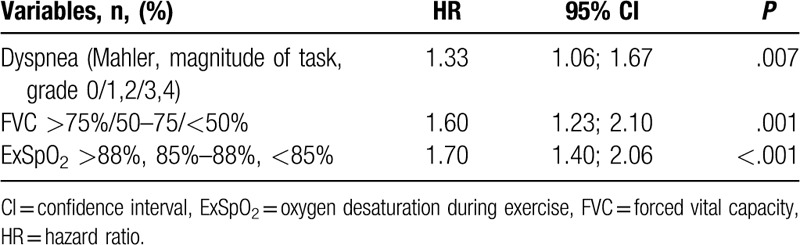
COX multivariate analysis for categorical dyspnea, functional variables, and ExSpO_2._

The treatments previously prescribed (none, corticosteroids, or immunosuppressors) did not influence the prognosis by multivariate analyses.

Data related to survival were categorized according to the best cutoffs, following several simulations and published data. The cut-offs for FVC% suggested by the GAP (gender, age, physiology) score (>75% predicted, 50%–75%, and <50%) were selected as the best prediction for survival.

By aggregating dyspnea in 3 categories, by Kaplan–Meier curves, the best separation was by holding dyspnea absent in a separate category, merging dyspnea with great and moderate efforts in a second category, and merging dyspnea with small efforts and at rest in a third category. By Kaplan–Meier curves, these categories showed significant median differences with 95% CIs without overlap.

Similarly, by Kaplan–Meier curves, ExSpO_2_ >88%, ExSpO_2_ 85% to 88%, and ExSpO_2_ <85% showed significant median differences in survival, with little overlap. Gender (*P* = .30), age (*P* = .89), and duration of symptoms did not relate to survival.

A Cox multivariate analysis was repeated with these 3 variables classified in 3 categories each, all contributing significantly to the model.

Based on described cut-offs, points were given to FVC >75% (0 point), 50% to 75% (1 point), and <50% (2 points); ExSpO_2_ >88% (0 point), 85% to 88% (1 point), and <85% (2 points); dyspnea absent (0 point), dyspnea to great and moderate efforts (1 point), and dyspnea to small efforts and at rest (2 points).

**Figure 1:**
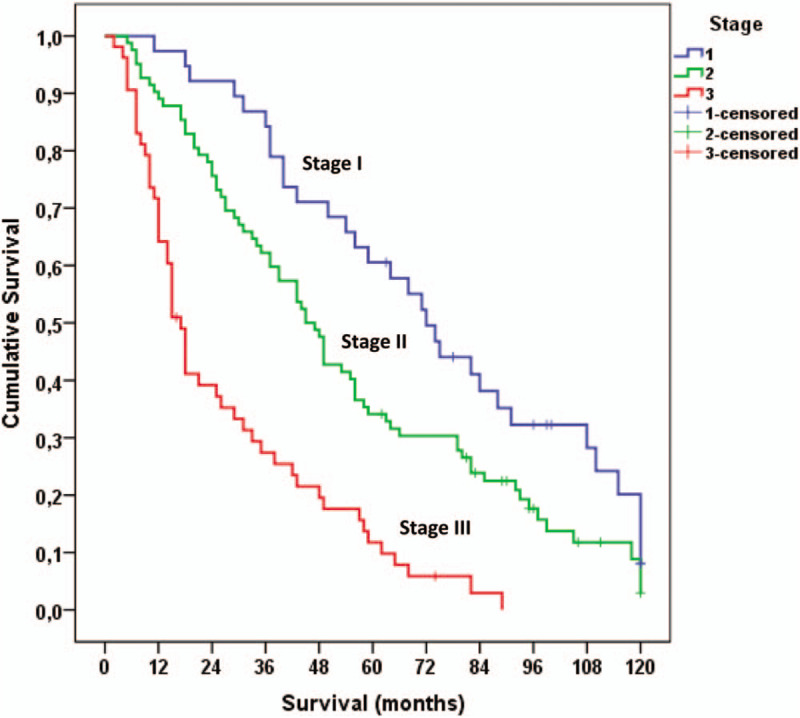
Survival in patients with IPF according to stages. IPF = idiopathic pulmonary fibrosis.

Survival was compared by adding points from these categories (0–6 points). A final stage was created by merging groups with similar survival curves: Stage 1 (n = 48), with 0/1 points, median survival 64 months (95% CI: 49–79 months); stage 2 (n = 72), with 2/3 points, median survival 45 months (95% CI: 37–53 months); and stage 3 (n = 53), with ≥4 points, median survival 17 months (95% CI: 14–20 months), log rank = 45.1, *P* < .001 (Table 4). The survival curves for the 3 groups are shown in Figure 1.

**Table 4:**
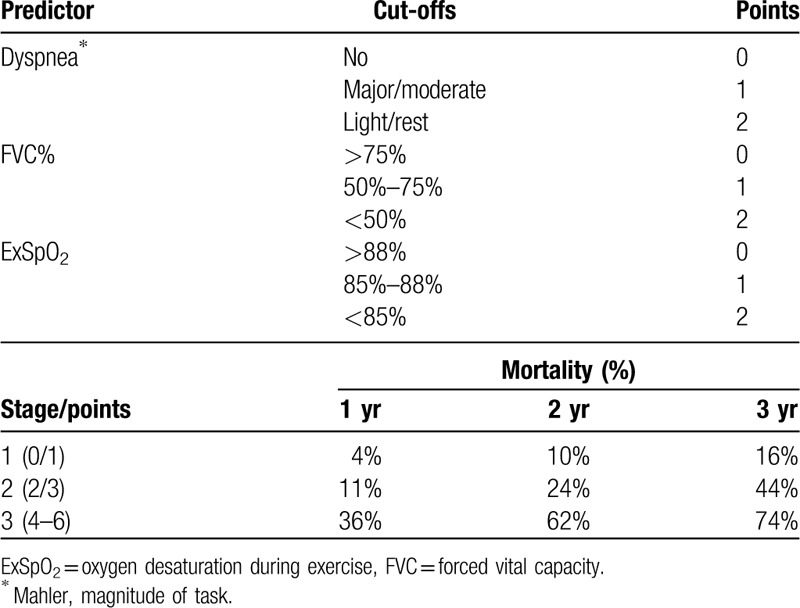
The DOS score (dyspnea, oxygen, and spirometry).

Thirteen patients had rest SpO_2_ <89% (use of O_2_). The median survival in this group was 15.0 months (95% CI: 8.1–21.9) compared to the remaining cases, in which median survival was 43 months (95% CI: 36.2–49.8), log rank = 15.66, *P* < .01.

The C-index for the score was 70.0 (95% CI: 59.0–80.0, *P* = .005).

The HR produced from the 10,000 bootstrap samples were similar to those of the original models, suggesting good internal validation (ExSpO_2_ = 1.60, 95% CI: 1.33–1.96, *P* < .001; dyspnea = 1.37, 95% CI: 1.05–1.83, *P* = .022; FVC = 1.60, 95% CI: 1.23–2.11, *P* < .001).

## 4 Discussion

In this study, we found that 3 physiological variables commonly measured during the initial evaluation of patients with IPF can predict mortality.

The development of a prognostic scoring system for IPF is important because it may serve as a basis for clinical decision making and simplify clinical trial design.^[8,9]^ Several studies have suggested a median survival of 2 to 4 years from the date of diagnosis of IPF. In our study, the median survival was 3.6 years.

Many individual clinical variables have been shown to predict survival in IPF.^[1,2]^ IPF is more prevalent in older males. In a GAP study,^[9]^ age and gender were significantly correlated with survival. In our study, age and gender did not relate to survival. Although some studies have found a worse prognosis in older individuals,^[4,8,9]^ others have found no influence.^[5–7,10,14,20]^ Similarly, a worse prognosis has been observed for males in some studies^[4,7,9]^ but not in others.^[5,6,8,14,20]^

General mortality was considered in a majority of studies on survival with IPF, and most deaths (approximately 80%) in IPF result from progression of lung fibrosis rather than from other causes.^[3]^ In the general population, mortality is greater in older men.^[23]^ This finding could explain the significant influence of age and gender on mortality in large study series of IPF.^[8,9]^

Concerning FVC, several cut-off points have been proposed, with lower FVC% values showing progressively greater HRs for mortality.^[8,9,24]^ In our study, cut-off points of FVC >75%, 50% to 75%, and < 50% were found to be the best discriminatory values. These cut-off points were similar to the GAP study.^[9]^

The D_L_CO is the functional variable that best correlates with disease extent in IPF,^[25]^ and it is the variable that is most reliably predictive of survival at baseline.^[2,3]^ The threshold of 40% was suggested by various authors.^[1–3,20]^ The D_L_CO measurements; however, can vary according to the type of equipment used, are not widely available, especially in developing and poor countries, and patients with severe lung function may not be able to perform the test.

Wallaert et al compared measurements of D_L_CO, resting PaO_2_, P(A-a) O_2_ at cardiopulmonary exercise testing peak and oxygen desaturation during a 6 MWT in 121 patients with IPF, and fibrotic nonspecific interstitial pneumonia, and showed a significant (*r* = –0.47), but moderate correlation between D_L_CO and oxygen desaturation during the 6 MWT.^[26]^

In the GAP study, oxygen use was removed from consideration because it had substantially different effects in the derivation and validation cohorts.^[9]^ In our study, similar to Sharp study, patients’ use of oxygen (SpO_2_ <89%) clearly had a poorer survival prognosis.

In a recent study,^[27]^ Rantala analyzed 44 patients with ILD with use of long-term oxygen therapy and found a median survival of 0.9 years.^[28]^

Previous studies showed a worse significant influence of oxygen desaturation, less than 89%, at the end of exercise on survival with IPF.^[14,15]^ Sharp et al demonstrated that exercise testing variables, including exertional desaturation, are good markers for early poor outcome and performed as consistently as multidimensional indices such as composite physiologic index (CPI) and GAP scores.^[27]^ In our study, ExSpO_2_ was the best predictive factor for survival by multivariate analysis.

In our study, by univariate analysis, FEV_1_/FVC >0.89 was associated with worse survival, an expected finding reflecting a higher degree of fibrosis.^[7,29]^ In IPF, the interpretation of lung function tests is confounded by coexistent emphysema, which results in spurious preservation of lung volumes, a lower FEV_1_/FVC, and worse gas transfer.^[30]^ In the present study, emphysema had no significant influence on survival, which has also been described by others.^[31]^ However, patients with an FEV_1_/FVC <0.70 were excluded.

Dyspnea is the most important factor influencing the quality of life of patients with IPF. As in other studies, we found that dyspnea has a significant and independent role in predicting survival.^[6,10,30]^ Exertion dyspnea and reduced exercise tolerance in IPF are multifactorial, and their correlations with functional variables are poor.^[32]^

Composite scoring systems have been developed that use physiological and radiographic variables to provide more accurate prognostic information about IPF.^[10,20,25]^ The extent of fibrosis on HRCT has great value when estimating prognosis. It can be more accurately measured by computational models, but these are available only in research centers.^[33]^

Other scores have been developed to estimate survival in IPF. The most cited are the CPI, which was developed using FEV_1_, FVC, and D_L_CO to predict the extent of disease on HRCT,^[25]^ and a multidimensional score (GAP), which included gender (G), age (A), and 2 lung physiology variables (FVC and D_L_CO) in the final model that was derived and validated.^[9]^ The CPI was a stronger predictor of mortality than individual measures of lung function such as FEV_1_, FVC, and D_L_CO.^[25]^ A CPI greater than 41 was predictive of worse survival (HR = 5.36) in IPF in a previous study.^[10]^ It is unclear whether it is possible to separate patients with high, intermediate, and low mortality with cut-off points derived from the CPI. Moreover, the score must be calculated from other parameters and is; therefore, not easy to apply in everyday clinical practice.

Even so, the GAP score showed mortality similar to our study. In 2 years, for example, estimated mortality was 10% versus 14% for stage I, 24% for both on stage II, and 62% for both scores on stage III.

Some limitations of our study should be stressed. First, the study was retrospective, but all deaths, except 3, were related to IPF or its complications. Several patients with IPF developed fatal acute exacerbation of the disease, so a completely reliable score for survival prediction is nearly impossible to obtain using baseline data.^[1]^ The use of categorical variables instead of continuous measurements is less desirable in prediction models, although it allows for simpler estimate scoring.

Some key factors should be considered when developing risk prediction models.^[34]^ The model must be validated in other cohorts; thus, our results must be replicated in other studies. The model should be able to discriminate those with an outcome from those without and should have clinical utility. The discrimination power of our model was calculated by the C-statistic, and the value was 70. The C-statistic ranges from 0.5 (model discrimination is no better than chance) to 1 (model discrimination is perfect). A C-statistic between 0.70 and 0.80 is considered acceptable.

The strengths of this study include an adequate sample size and a substantial follow-up duration with a high rate of mortality, enough for analyzing the role of the selected predictor variables.^[18]^

This model will help physicians establish the ideal time for lung transplantation, randomize patients with similar prognosis in clinical trials, and contribute factors for discussion of prognosis with patients and relatives.

We examined a well-characterized population of patients with IPF and developed a prognostic score with easily measured variables, including dyspnea, and percentage-predicted FVC and ExSpO_2_. Categorical values for these variables can be combined to derive a score that is predictive of high, intermediate, and low mortality.

## Author contributions

**Conceptualization:** Carlos Alberto de Castro Pereira.

**Formal analysis:** Carlos Alberto de Castro Pereira.

**Investigation:** Cesar Yoshito Fukuda, Maria Raquel Soares.

**Methodology:** Cesar Yoshito Fukuda, Carlos Alberto de Castro Pereira.

**Project administration:** Cesar Yoshito Fukuda, Carlos Alberto de Castro Pereira.

**Supervision:** Carlos Alberto de Castro Pereira.

**Validation:** Cesar Yoshito Fukuda, Carlos Alberto de Castro Pereira.

**Writing – original draft:** Cesar Yoshito Fukuda, Maria Raquel Soares, Carlos Alberto de Castro Pereira.

**Writing – review & editing:** Cesar Yoshito Fukuda, Maria Raquel Soares, Carlos Alberto de Castro Pereira.
